# The Protective Effect of Naringin against Bleomycin-Induced Pulmonary Fibrosis in Wistar Rats

**DOI:** 10.1155/2016/7601393

**Published:** 2016-02-10

**Authors:** Nergiz H. Turgut, Haki Kara, Sahende Elagoz, Koksal Deveci, Huseyin Gungor, Emre Arslanbas

**Affiliations:** ^1^Cumhuriyet University School of Pharmacy, Department of Pharmacology, 58140 Sivas, Turkey; ^2^Cumhuriyet University School of Veterinary Medicine, Department of Pharmacology and Toxicology, 58140 Sivas, Turkey; ^3^Cumhuriyet University School of Medicine, Department of Pathology, 58140 Sivas, Turkey; ^4^Cumhuriyet University School of Medicine, Department of Biochemistry, 58140 Sivas, Turkey

## Abstract

The aim of the current study was to investigate the protective effect of naringin on bleomycin-induced pulmonary fibrosis in rats. Twenty-four Wistar rats randomly divided into four groups (control, bleomycin alone, bleomycin + naringin 40, and bleomycin + naringin 80) were used. Rats were administered a single dose of bleomycin (5 mg/kg; via the tracheal cannula) alone or followed by either naringin 40 mg/kg (orally) or naringin 80 mg/kg (orally) or water (1 mL, orally) for 14 days. Rats and lung tissue were weighed to determine the lung index. TNF-*α* and IL-1*β* levels, hydroxyproline content, and malondialdehyde (MDA) levels were assayed. Glutathione peroxidase (GSH-Px) and superoxide dismutase (SOD) activities were determined. Tissue sections were stained with hematoxylin-eosin, Masson's trichrome, and 0.1% toluidine blue. TNF-*α*, IL-1*β*, and MDA levels and hydroxyproline content significantly increased (*p* < 0.01) and GPx and SOD activities significantly decreased in bleomycin group (*p* < 0.01). Naringin at a dose of 80 mg/kg body weight significantly decreased TNF-*α* and IL-1*β* activity, hydroxyproline content, and MDA level (*p* < 0.01) and increased GPx and SOD activities (*p* < 0.05). Histological evidence supported the results. These results show that naringin has the potential of reducing the toxic effects of bleomycin and may provide supportive therapy for conventional treatment methods for idiopathic pulmonary fibrosis.

## 1. Introduction

Idiopathic pulmonary fibrosis (IPF) is a progressive and devastating lung disease characterized by deposition of extracellular matrix which leads to lung remodeling. The disease is thought to be driven by abnormal and/or dysfunctional alveolar epithelial cells which support fibroblast proliferation, recruitment, and differentiation [1]. Early and accurate diagnosis of the disease is important. From the time of diagnosis, the average survival time is 2.8–4.2 years which is worse than some types of cancer [2]. Idiopathic pulmonary fibrosis mostly occurs after the age of sixty and is more common in men. Pulmonary fibrosis progresses slowly and insidiously for many patients; however, approximately in 10–15% of patients, the course of the disease is much more rapid and the acute exacerbation of the disease is a highly lethal clinical event [3, 4]. Dry cough, chronic exertional dyspnea, bibasilar end-inspiratory crackles, and finger clubbing are clinical features commonly observed with IPF. Medications used in treatment such as steroids and immunosuppressive drugs are insufficient to improve prognosis [5, 6]. Therefore it is necessary to develop new types of drugs.

The etiology of idiopathic fibrosis is yet not fully understood. Recent studies have suggested that lung injury can be induced by inflammatory cells through the release of oxidative species, cytokines, proteases, peroxidases, and growth factors [7, 8]. Although inflammation is not the primary mechanism of IPF, it may contribute to the disease pathogenesis by affecting the level of oxidative stress and wound-repair process. Reactive oxygen molecules (ROS) released from active leukocytes contribute to lung damage and inflammation. In some studies carried out recently, it has been suggested that a balance between ROS and antioxidant molecules plays an important role in the pathogenesis of pulmonary fibrosis [9, 10]. Increased levels of ROS have been reported in bronchoalveolar lavage fluid (BALF), obtained from the lung tissue of patients with pulmonary fibrosis and from bleomycin administered animals [11, 12]. It is well known that the accumulated macrophages and neutrophils are capable of inducing oxidant-mediated lung parenchyma cell toxicity [13]. Activated neutrophils can yield myeloperoxidase (MPO) and this enzyme can create toxic hydroxyl radicals by combining with hydrogen peroxide [14]. The supportive therapy provided by agents with both antioxidant and anti-inflammatory properties may be useful to treat IPF and other disorders characterized by excessive inflammation and/or oxidative stress.

Bleomycin is a widely used antineoplastic drug. However, because it creates dose dependent pulmonary fibrosis, the effective use of the drug in chemotherapy is limited. The binding of bleomycin to DNA and iron induces production of ROS and initiates inflammatory changes through action of cytokines that lead to collagen accumulation in the lung [15, 16]. To evaluate candidate drugs for pulmonary fibrosis, bleomycin-induced lung fibrosis animal model offers convenient option for study [3, 17].

Among present natural antioxidants, flavonoids are large in number and are one of the most common groups. They are potent inhibitors of lipid peroxidation in biological membranes and are widely distributed in vegetables, fruits, seeds, leaves, and barks of plants [18]. Flavonoids generally comprise one or more aromatic hydroxyl groups and these groups are responsible for the flavonoid antioxidant effect [19]. Naringin is a major and active flavone glucoside found in grape and many citrus fruits. When administered orally, it is hydrolyzed to its major metabolite olite-naringin by enzymes such as *α*-rhamnosidase and *β*-glucosidase [20]. Naringin has been reported to have various pharmacological and therapeutic properties such as antimicrobial, antimutagenic, chemoprotective, anticancer, inflammation relieving, and cholesterol lowering effects [21–23]. Growing evidence indicates that naringin has both in vivo and in vitro anti-inflammatory effects [20, 22]. Although the positive effect of naringin against different oxidative stress related diseases has been reported [24, 25], studies investigating its effect in pulmonary fibrosis are limited.

The aim of the present study was to investigate the possible protective effect and the mechanism of the therapeutic efficacy of naringin in rats with bleomycin-induced pulmonary fibrosis by comparing both oxidant-antioxidant and inflammatory status.

## 2. Materials and Methods

### 2.1. Drug and Chemicals

Naringin and the agents used for diagnostic purposes were obtained from Sigma Chemical Co. (St. Louis, MO, USA). Bleomycin was obtained from Onko Pharmaceutical Industry and Trade. Ltd. (Istanbul, Turkey). The kits for the determination of superoxide dismutase (SOD) and glutathione peroxidase (GPx) were purchased from Cayman Chemical Company (USA). The ELISA kit for hydroxyproline was purchased from Hangzhou Eastbiopharm Company Ltd. (China) and the ELISA kits for tumor necrosis factor alpha (TNF-*α*) and interleukin-1 beta (IL-1*β*) were purchased from eBioscience Company (USA). The purity of all chemical reagents was at least analytical grade.

### 2.2. Animals

In this study, a randomized experimental protocol was used. The study was carried out with 24 male Wistar-albino rats with an average body weight of 170–210 g. The rats were housed in standard laboratory conditions (12 h light/dark cycles, 24 ± 2°C, and 35–60% humidity) and fed with standard pellet chow and water ad libitum. The experiments were made in accordance with EU Directive 2010/63/EU for animal experiments. The study protocol was approved by Cumhuriyet University, Animal Care and Use Committee.

### 2.3. Induction of Pulmonary Fibrosis and Treatment Protocols

A total of 24 Wistar-albino rats, randomly divided into four groups (control, bleomycin alone, bleomycin + naringin 40, and bleomycin + naringin 80), were used in the study. After overnight fasting, the rats were anesthetized (60 mg/kg ketamine HCl and 5 mg/kg xylazine) and a midline incision was made in the neck and the trachea was exposed by blunt dissection. A tracheal cannula was inserted under direct visualization into the trachea. Pulmonary fibrosis was induced by intratracheal administration of single dose 0.1 mL of bleomycin (5 mg/kg in 0.9% NaCl) [26, 27]. The same procedure was performed for the control group with the exception that saline was substituted for bleomycin. In treatment groups after bleomycin administration, rats were treated orally (gavage) with 40 mg/kg/day or 80 mg/kg/day of naringin. Naringin doses 40 mg/kg/day and 80 mg/kg/day were similar to the doses used by previous studies in literature [28–30]. Naringin was given 5 min after administration of bleomycin for 14 consecutive days. After 14 days, BALF was obtained and then animals were decapitated to remove the lungs. The lungs were trimmed from the extraneous tissue and rinsed.

All rats were weighed before and at the end of the experiments. The changes in body weight were determined. Also lung tissue was weighed to determine the lung index. Lung index was determined by dividing lung weight (g) by body weight (g) and multiplying by 100. While one of the lobes was separated for histopathologic analysis, the second lobe was stored at −80°C for further analysis.

### 2.4. Estimation of Lipid Peroxidation

Lipid peroxidation was monitored in terms of malondialdehyde (MDA) by the method of Ohkawa et al. [31]. MDA level was determined by thiobarbituric acid reactive substances (TBARS) in lung tissue homogenate, based on the reaction between MDA and thiobarbituric acid. Thiobarbituric acid when allowed to react with MDA aerobically formed a colored complex [MDA - (TBA) 2 complex] which was measured by the spectrophotometer (Shimadzu UV-1700, Japan) at 532 nm. MDA concentration (measured as TBARS) was calculated as “nmol/mL.” Absorbance values were compared with a series of standard solutions of 1,1,3,3-tetraethoxypropane (TEP).

### 2.5. Activities of Enzymatic Antioxidants

Superoxide dismutase was determined by using commercially available standard enzymatic kit (Cayman Chemical Company, USA). Lung tissue was homogenized in cold 20 mM HEPES buffer (pH 7.2) containing 1 mM EGTA, 210 mM mannitol, and 70 mM sucrose per gram tissue and was centrifuged at 1500 ×g for 5 min at 4°C. The supernatant was removed and stored on ice for total SOD activity (cytosolic and mitochondrial) assay according to the protocol provided by the assay kit manufacturer. The absorbance was read at 440–460 nm using a plate reader (Thermo Scientific Multiskan GO Microplate Spectrophotometer, USA). The SOD activity was expressed as U/mg protein. This kit utilizes a tetrazolium salt for the detection of superoxide radicals generated by xanthine oxidase and hypoxanthine. The SOD assay measures all three types of SOD (CU/Zn, Mn, and Fe SOD). One unit of SOD is defined as the amount of enzyme needed to exhibit 50% dismutation of the superoxide radical.

Glutathione peroxidase activity was measured by using a commercially available kit (Cayman Chemical, USA). Lung tissue was homogenized in 50 mM Tris-HCl buffer (pH 7.5) containing 5 mM EDTA and 1 mM DTT per gram tissue and centrifuged at 10,000 ×g for 15 min at 4°C to obtain supernatant for GPx analysis. The removed supernatant was stored on ice and the absorbance was read once every minute at 340 nm using a plate reader to obtain at least 5 time points. Levels of GPx activity were expressed as nmol/min/mg protein in tissue. The measurement of GPx activity is based on the principle of a coupled reaction with glutathione reductase (GR). The oxidized glutathione (GSSG) formed after the reduction of hydroperoxide by GPx is recycled to its reduced state by GR in the presence of NADPH and is accompanied by a decrease in absorbance at 340 nm. One unit of GPx is defined as the amount of enzyme that catalyzes the oxidation of 1 nmol of NADPH per minute at 25°C. The total protein content was estimated by the method of Lowry et al. [32].

### 2.6. Hydroxyproline Assay

Hydroxyproline assay was performed by using a commercially available ELISA kit (Hangzhou Eastbiopharm Co., Ltd., China). Lung tissue was homogenized and centrifuged for 5 minutes at 4°C, 1500 ×g. The supernatant was separated and stored on ice. Absorbance was read at 450 nm using a plate reader. Hydroxyproline levels were expressed as *μ*g/right lung.

### 2.7. Measurement of TNF-*α* and IL-1*β* Levels

TNF-*α* and IL-1*β* levels were measured by using commercially available kits (eBioscience Co., USA). BALF was centrifuged at 4°C, 1200 ×g for 10 minutes. The supernatant was separated and stored on ice. Absorbance was read at 450 nm using a plate reader. TNF-*α* and IL-1*β* levels were expressed as pg/mL.

### 2.8. Histopathological Examinations

The lung tissues from all groups of animals were fixed in 10% formaldehyde and processed routinely for embedding in paraffin. Serial sections were cut. Tissue sections (4 *μ*m thick) were stained with hematoxylin and eosin and also with Masson's trichrome. Tissue sections were examined under light microscope by taking photomicrographs (Nikon Eclipse 80, USA). The severity of pulmonary fibrosis was evaluated by Ashcroft grading system [33]. According to the severity of inflammation, scoring was performed as follows: +/++/+++. The histopathologic evaluation was performed by an experienced pathologist, unaware of the treatment groups.

The grade of lung fibrosis was scored on a scale of 0 to 8. Criteria for scoring pulmonary fibrosis were as follows: Grade 0 = normal lung; Grade 1 = minimal fibrous thickening of alveolar or bronchiolar walls; Grades 2-3 = moderate thickening of walls without obvious damage to lung architecture; Grades 4-5 = increased fibrosis with definite damage to lung architecture and formation of fibrous bands or small fibrous mass; Grades 6-7 = severe distortion of structure and large fibrous areas; Grade 8 = total fibrotic obliteration.

### 2.9. Mast Cell Analysis

In order to show mast cells, lung tissue sections were stained with 0.1% toluidine blue as previously described [34]. To record average mast cells, in 10 high magnification random fields, photomicrographs (Nikon Eclipse 80i, USA) were taken and positive stained cells with toluidine blue were counted.

### 2.10. Statistical Analysis

Data were analyzed by SPSS for windows, version 10 (SPSS Inc., Chicago, IL, USA). The data were expressed as means ± S.D. and analyzed using one-way ANOVA followed by Tukey-Kramer multiple comparison tests. Previously, the normal distribution of data was evaluated. Differences were considered statistically significant at *p* < 0.05.

## 3. Results

### 3.1. Effect of Naringin on Body Weight and Lung Index

**Figure 1:**
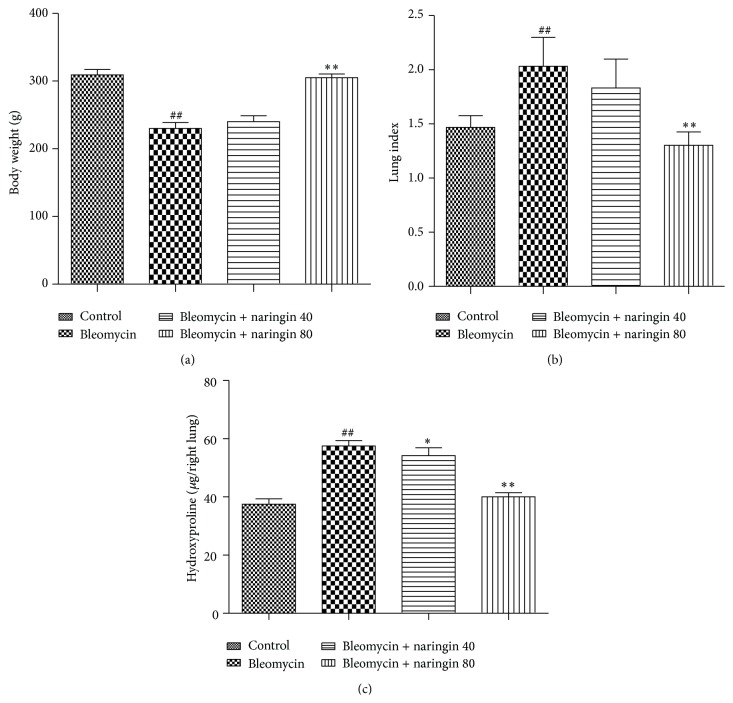
(a) Effect of naringin on body weight in rats with pulmonary fibrosis. (b) Effect of naringin on lung index in rats with pulmonary fibrosis. (c) Hydroxyproline levels of lung tissue in bleomycin, naringin, and control groups. Data are given as mean ± S.D. from six rats in each group. ^##^ *p* < 0.01, compared with the control group; ^*∗*^ *p* < 0.05, ^*∗∗*^ *p* < 0.01 compared to bleomycin group. For statistical analysis, one-way ANOVA test followed by Tukey-Kramer multiple comparison test was used.

Treatment with bleomycin resulted in marked decrease in body weight compared to control group (*p* < 0.01). Administration of naringin (80 mg/kg) led to a significant increase in body weight as compared to the bleomycin group (*p* < 0.01) (Figure 1(a)). On the other side, treatment of rats with bleomycin showed an increase in lung index as compared to control group (*p* < 0.01). Naringin (80 mg/kg) administration led to a marked decrease in lung index compared with the bleomycin group (*p* < 0.01) (Figure 1(b)). Naringin (40 mg/kg) showed no significant change in body weight and lung index compared to bleomycin group.

### 3.2. Effect of Naringin on Hydroxyproline Levels

Collagen deposition in lung tissues was determined by measuring the hydroxyproline content. Compared with the control group, hydroxyproline content was increased significantly in rat lung in the bleomycin group (*p* < 0.01). The increased hydroxyproline content significantly reduced dose dependently with naringin (40 and 80 mg/kg) administration (*p* < 0.05, *p* < 0.01) (Figure 1(c)).

### 3.3. Lipid Peroxidation Levels

MDA levels significantly increased in bleomycin treated rats compared to control group (*p* < 0.01). Naringin administration at 80 mg/kg to bleomycin treated rats significantly lowered the MDA levels compared to bleomycin group (*p* < 0.01); whereas 40 mg/kg did not cause a change in MDA levels significantly (Table 1).

### 3.4. Effect of Naringin on Enzymatic Antioxidants

A significant decrease (*p* < 0.01) in the levels of SOD and GPx was noticed in rats treated with bleomycin when compared to control rats. Treatment of naringin (80 mg/kg) in bleomycin administered rats increased the levels of SOD and GPx significantly compared to bleomycin group (*p* < 0.05) (Table 1).

### 3.5. Effect of Naringin on TNF-*α* and IL-1*β* Levels

**Figure 2:**
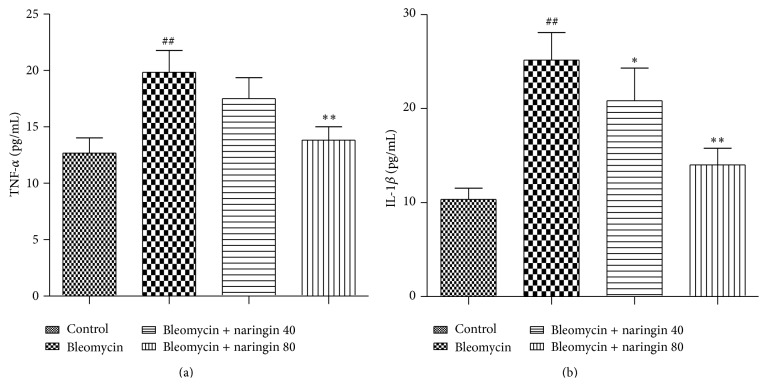
Tumor necrosis factor alpha (TNF-*α*) (a) and interleukin-1 beta (IL-1*β*) (b) levels in bronchoalveolar lavage fluid (BALF) of bleomycin, naringin, and control group rats. Data were expressed as mean ± S.D. ^##^ *p* < 0.01, compared to the control group; ^*∗*^ *p* < 0.05, ^*∗∗*^ *p* < 0.01 compared to bleomycin group. For statistical analysis, one-way ANOVA test followed by Tukey-Kramer multiple comparison test was used.

BALF and proinflammatory cytokine levels remained elevated in the bleomycin group compared to control groups (*p* < 0.01 both for TNF-*α* and IL-1*β*). Naringin (80 mg/kg) application was effectively able to attenuate the elevation of TNF-*α* (*p* < 0.01) and both doses of naringin were able to decrease the levels of IL-1*β* levels in a dose dependent manner compared to bleomycin group (*p* < 0.05; *p* < 0.01) (Figure 2).

### 3.6. Histopathological Changes

**Figure 3:**
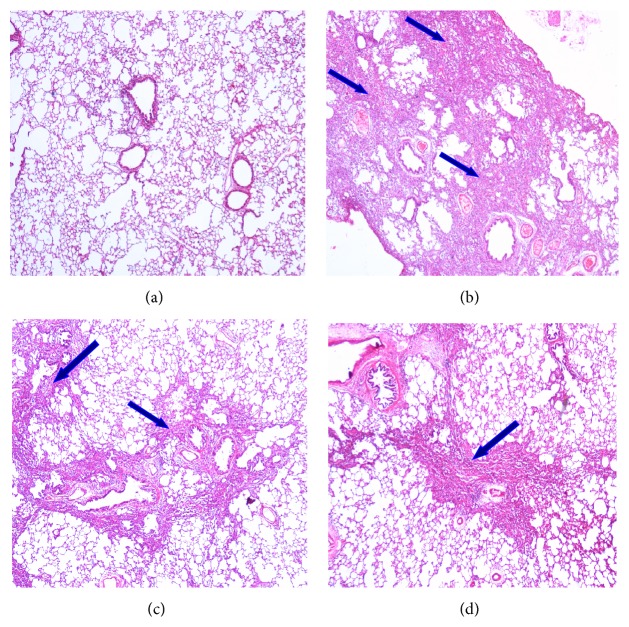
(a) The hematoxylin and eosin staining was carried out according to the regular staining method. (a) Control group: normal appearance of lung parenchyma; (b) bleomycin group: significant infiltration of inflammatory cells (+++); (c) bleomycin + naringin 40 group: moderate infiltration of inflammatory cells in the tissue (++); (d) bleomycin + naringin 80 group: a small amount of inflammatory cell infiltration in the tissue (+) (HE ×40). Arrows show areas with inflammatory cells.

**Figure 4:**
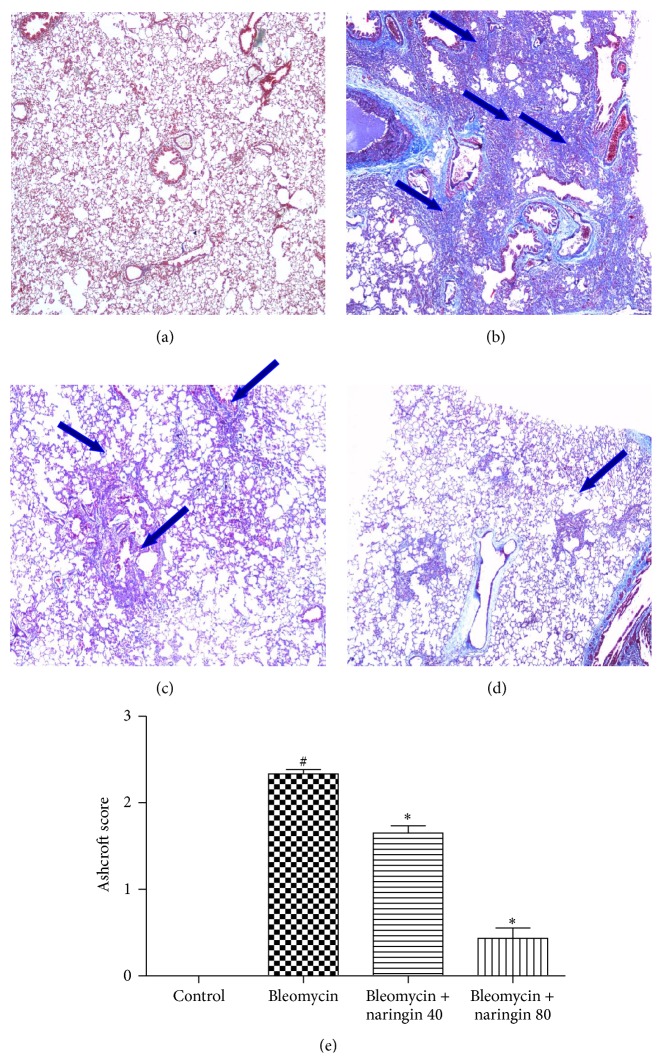
Photomicrographs of Masson's trichrome stained lung tissues from each group. (a) Control group: normal appearance of lung parenchyma (Grade 0); (b) bleomycin group: severe distortion of structure and large fibrous areas (Grades 6-7); (c) bleomycin + naringin 40 group: increased fibrosis with definite damage to lung architecture and formation of fibrous bands (Grades 4-5); (d) bleomycin + naringin 80 group: minimal fibrous thickening of alveolar or bronchiolar walls (Grade 1) (MT ×40). (e) The quantitative evaluation of fibrotic changes in control, bleomycin, naringin 40, and naringin 80 groups by Ashcroft scoring. ^#^ *p* < 0.05, compared with control group; ^*∗*^ *p* < 0.05; compared with bleomycin group. Arrows show areas with fibrotic changes. For statistical analysis, one-way ANOVA test followed by Tukey-Kramer multiple comparison test was used.

For control and experimental groups, histopathological photographs of lung tissue stained with hematoxylin and eosin are shown in Figure 3. Lungs of control rats showed normal lung morphology with normal alveolar spaces and normal thickening of alveolar septa (Figure 3(a)). The bleomycin treatment led to abnormal morphologies including significant interstitial infiltration by inflammatory cells, alveolar septal thickening, and collapsed alveolar spaces (Figure 3(b)). Naringin treatments showed protection against bleomycin-induced lung damage in a dose dependent manner (Figures 3(c) and 3(d)). In naringin (80 mg/kg) treated group, significant decrease in cellular infiltrates and thin lined alveolar septa were observed compared with bleomycin-induced animals (Figure 3(d)). Inflammation scoring was performed depending on the severity of inflammation as follows: +/++/+++. In bleomycin group, significant infiltration of inflammatory cells (+++); in bleomycin + naringin 40 group, moderate infiltration of inflammatory cells (++); and, in bleomycin + naringin 80 group, a small amount of inflammatory cell infiltration (+) were observed in the lung tissue (Figure 3). For control and experimental groups, histopathological photographs of lung tissue stained with Masson's trichrome are shown in Figure 4. For assessing the degree of pulmonary fibrosis, Ashcroft grading system was used. Normal lung histology was seen in control group rats (Grade 0) (Figure 4(a)). Naringin (80 mg/kg) treated group revealed that naringin had a moderate protective effect on pulmonary injury (Grade 1) (Figure 4(d)). Masson staining of lung specimens showed that bleomycin induced severe distortion of structure and accumulation of collagen fiber in rat lungs (Grades 6-7) (Figure 4(b)). The Ashcroft quantitative pathological scoring is presented in Figure 4(e). The score was high in bleomycin group compared to control group animals. Naringin treatment (40 and 80 mg/kg) significantly reduced the score compared to bleomycin group (*p* < 0.05).

### 3.7. Effect of Naringin on Mast Cells

**Figure 5:**
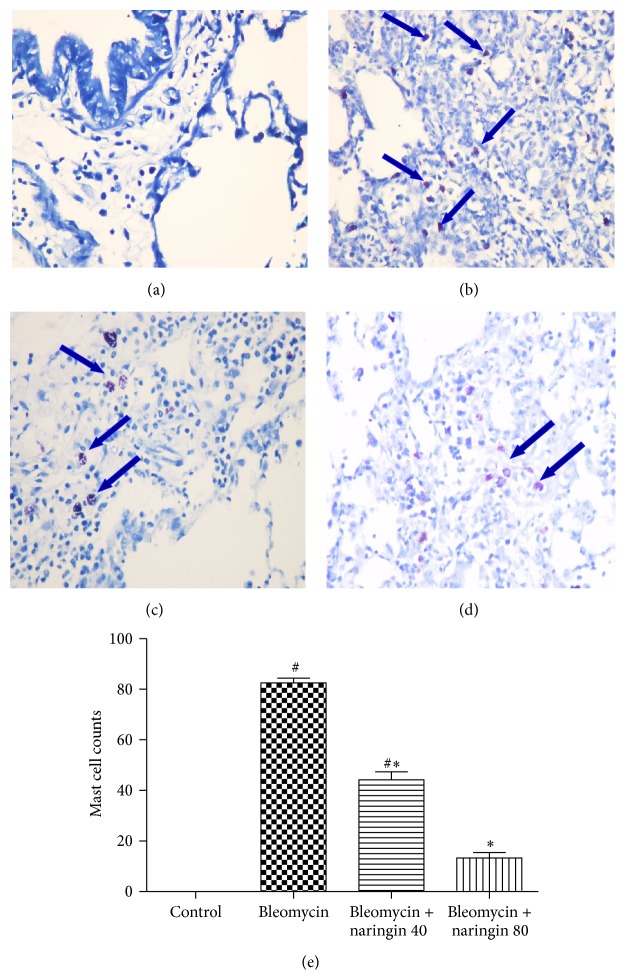
Toluidine blue staining for mast cell analysis. (a) Control group: no mast cell was seen; (b) bleomycin group: dense mast cell accumulation; (c) bleomycin + naringin 40 group: reduced mast cells; (d) bleomycin + naringin 80 group: considerably reduced mast cells; (e) bar graph shows the quantitative mast cell counts in each group of animals. Data are given as mean ± S.D. from six rats in each group, ^#^ *p* < 0.05, compared with control group; ^*∗*^ *p* < 0.05, compared with bleomycin group. Arrows show mast cells stained with toluidine blue. For statistical analysis, one-way ANOVA test followed by Tukey-Kramer multiple comparison test was used.

Tissue sections were stained with toluidine blue to analyze the mast cell mediated fibrogenic events. In bleomycin-induced group, high level of mast cell populations were observed (Figure 5(b)), whereas no mast cell was seen in control group of animals (Figure 5(a)). Control group animals showed no significant differences in the mast cell recruitments. Naringin administration reduced the mast cell recruitments as seen in Figures 5(c) and 5(d). Naringin 80 mg/kg effect was more prominent. The mean number of mast cells across 10 random fields for each group is represented in Figure 5(e).

## 4. Discussion

Idiopathic pulmonary fibrosis is a lung disorder of unknown etiology. The underlying mechanisms are widely studied but it is accepted that cytokine mediated injuries and airway inflammatory cell accumulations contribute to the fibrotic progression [4]. To our knowledge, there is no report studying the effect of naringin administration against bleomycin-induced pulmonary fibrosis. Therefore the aim of the present study was to investigate the possible protective effect and the mechanism of the therapeutic efficacy of naringin in rats with bleomycin-induced pulmonary fibrosis by comparing both oxidant-antioxidant and inflammatory status. The data obtained from the study demonstrated the protective effect of naringin (80 mg/kg, body weight) treatment against proinflammatory cytokines, oxidative stress, and histological changes.

In this study, the most commonly used rodent model, bleomycin model, involving intratracheal instillation of bleomycin was used [3, 35]. The enzyme bleomycin hydrolase inactivates bleomycin, and as lung produces this enzyme in lower levels, it is more susceptible to the damaging toxic effects of bleomycin. The agent can be administered in a single dose into the airway by intratracheal route and, within a short period of time, this leads to inflammatory and fibrosis manifestations [36]. After drug administration, initial elevation in cytokines such as TNF-*α* and IL-1*β* occurs. This is followed by increased expression of the profibrotic cytokine transforming growth factor *β* (TGF-*β*). Despite being an easy referenced model resembling acute lung injury in some way, the development of fibrosis may be partially reversible in this model [37]. As the pathophysiology of bleomycin infusion includes high oxidative stress and inflammation, we found the use of this model suitable for investigating the protective effect of naringin which has anti-inflammatory and antioxidant properties.

Fibrotic phases are biphasic and early inflammatory and late fibrotic phases. After bleomycin administration, these phases become apparent at the 14th day and produce the maximum peak at the 28th day. As the profibrogenic molecules are generated along with inflammation in the early phase (0–14 days), test agents applied in this phase are considered protective [38]. From this point, in our study naringin treatments were provided to experimental animals for 14 days with a protective approach.

Body weight decrement is among symptoms of bleomycin toxicity. Treatment with bleomycin caused a significant weight loss and a significant lung index increase in rats. In bleomycin applied rats, the changes in organ-body weight and the decrease in body weight may be associated with the acute injury in fibrosis occurring in lung [39]. Naringin applications increased body weight while these applications decreased organ-body weight (index of lung).

Bleomycin is known to cause oxidative damage in the lungs [3]. Increased lipid peroxidation is a typical result of ROS. In the inflamed tissue, there are several sources for ROS, including epithelium, activated inflammatory cells, and/or microvascular endothelium. Recent studies have demonstrated that fibroblasts can derive from the lung epithelium through epithelial-mesenchymal transition (EMT) and that they contribute to fibrosis in experimental animal IPF models [40–42]. Previously performed studies with single dose bleomycin model suggest that about one-third of the fibroblasts are derived from EMT throughout experimental pulmonary fibrosis [43, 44].

Malondialdehyde is a reactive carbon compound which is used as an indicator of lipid peroxidation [45]. Consistent with previous studies [46, 47], there was a significant increase in MDA levels in the lung tissue of bleomycin administered rats. This proves the involvement of ROS mediated lung injury in the pathogenesis. Scavenging of free radicals seems to have an important role in the antioxidant activity of flavonoids. The relation between the chemical nature of flavonoids and their antioxidant activity has been determined. This antioxidant activity refers to the phenolic hydroxyl groups attached to the flavonoid structure [19, 22]. Naringin administration significantly reduced bleomycin-induced oxidative stress as evidenced from the decreased levels of MDA. This protective effect of naringin is thought to be connected to its free radical scavenger effect and antioxidant activity, decreasing oxidative stress caused by bleomycin via generation of ROS.

Endogenous enzymes such as SOD and GPx play an important role in the cellular protection system against oxidative damage. These endogenous antioxidant enzymes eliminate reactive oxygen species such as hydrogen peroxide and prevent hydroxyl radical formation [48]. Rising increased SOD activity results in the removal of superoxide radical; thus the pulmonary damage generated by free radicals decreases. The balance between free radicals and antioxidants is important. In pathological conditions, the formation of reactive oxygen disrupts this balance [49]. In our study, decrease in antiperoxidative enzymes, SOD and GPx, was observed in bleomycin treated rats. The decrease in GPx level in bleomycin treated rats can be related to the reduction of glutathione. The observed significant decrease in antioxidant levels of bleomycin-induced animal group is the result of overproduction of ROS [48, 50]. Naringin treatment increased SOD and GPx activity and this case may be associated with decreasing formation of hydroxyl radical and superoxide radical scavenger effect of naringin. This protective effect might also be mediated by increased synthesis of glutathione that is believed to have intracellular protective effect. Similar results have been reported [51, 52]. Our results showing the decrease in MDA levels along with the increase in SOD and GPx activity imply that naringin is beneficial in maintaining oxidant-antioxidant balance.

Bleomycin is known to produce free radicals such as superoxide and hydroxyl radical, and the synthesis of collagen in the lungs increases with these radicals. After bleomycin administration, cytokine dysregulation and inflammation develop, fibroblasts are activated, and collagen production is stimulated while collagen degradation is inhibited [53]. In our study, hydroxyproline content which is an indicator of collagen deposition increased by bleomycin treatment and with naringin treatment it reduced significantly. This effect of naringin can be explained with possible mechanisms such as inhibiting lung inflammatory cell accumulation and thus reducing free oxygen radical production, removal of present free radicals from environment, detoxifying free radicals generated by bleomycin, and thus inhibition of fibroblast proliferation.

Growing evidences strongly show the essential role of cytokines in the pathogenesis of pulmonary fibrosis [54, 55]. These cytokines have overlapping and synergetic activities inducing the production of other cytokines, arachidonic acid metabolites, and adhesion molecules and they also activate immune and nonimmune cells. Increased expression of TNF-*α* and IL-1*β* has been found in the lungs of patients and animal models of pulmonary fibrosis [2, 5, 19]. TGF-*β* is a potent profibrogenic cytokine that is upregulated in lungs of patients with IPF. This cytokine regulates tissue morphogenesis and differentiation and induces a fibrotic response in many tissues including lung [56, 57]. When bleomycin-induced pulmonary fibrosis starts producing, sudden inflammatory responses occur which induce inflammatory cells and increase the number of macrophages [7, 58]. These cytokines are associated with airway fibrosis, due to their ability to regulate the formation of fibroblasts and matrix [59]. Naringin was also reported to reduce inflammation by inhibiting NF-*κ*B activity in a mice lipopolysaccharide induced acute lung injury model [60]. In our study, naringin reduced upregulation of proinflammatory cytokines: TNF-*α* and IL-1*β*. These results indicate that anti-inflammatory effect of naringin on bleomycin-induced pulmonary fibrosis may depend on a decrease in the production of proinflammatory cytokines.

Histopathological evaluation supported the results obtained in the study. In agreement with previous reports, the histological signs of bleomycin-induced group of animals showed high score values on Ashcroft scoring [26, 61]. Bleomycin administration induced inflammatory cell infiltrate which may be due to the formation of reactive radicals produced by the oxidative threat induced by bleomycin. The histopathology results of the study clearly show that naringin application attenuates the extent and severity of the histological signs of tissue damage. Naringin administration could suppress bleomycin-induced inflammatory cell infiltrate and collagen deposition which reduces the oxidative stress that leads to reduction of pathological changes. This can be attributed to the antioxidant and anti-inflammatory effects of naringin. Mast cells are specialized granulocytes which participate in wound-healing process. These cells have an important role in the early asthmatic reaction. Mast cell derived mediators induce bronchoconstriction, mucosal edema, and mucus secretion in early asthma stage. There is growing evidence that these cells may also play a role in the pathogenesis of other airway diseases including IPF [62]. Mast cells have been shown to stimulate the migration and proliferation of fibroblasts and mast cell hyperplasia is shown in fibrotic lung disorders. These cells can release preformed mediators including histamine and mast specific proteases which activate near fibroblasts to secrete fibrogenic cytokines that advance to fibrosis [63]. It has been shown that mast cells and the mast cell-specific chymase MCPT4 can mediate acute lung inflammation and injury in mice [64]. Compared to control group animals, in bleomycin-induced animals, an increased influx of mast cells was observed. Naringin applications significantly reduced mast cell recruitments. Chen et al. as well as our study also investigated the protective effects of naringin on pulmonary fibrosis caused by paraquat in a mice model and suggested that naringin may be a potential therapeutic for management of paraquat intoxication [52]. Since naringin is a flavonoid taken together with the diet in this study, we focused on investigating its protective efficacy on lung injury. Additional studies investigating the therapeutic effect of naringin at late fibrotic phases should be conducted. Also total and differential cell count in BALF and changes to TGF-*β* will be needed to explore in future research.

## 5. Conclusion

Our current study demonstrates the protective effects of naringin against bleomycin-induced acute lung inflammation and fibrosis in rats as confirmed by biochemical assays and histopathological evaluation. Naringin markedly improved lung morphology. A significant reduction in hydroxyproline and lung collagen content was measured. BALF, TNF-*α*, and IL-1*β* levels induced by bleomycin were also significantly suppressed by naringin. Naringin decreased lipid peroxidation and increased antioxidant defense enzyme levels. Naringin's protective effect was observed with doses of 80 mg/kg body weight. This protective effect of naringin may be due to its potential of reducing inflammatory cytokine levels, preventing the formation of oxygen free radicals, and/or removing them from the medium and also its antioxidant nature. Combining antioxidants or inhibitors of oxidant generation that also have anti-inflammatory properties with other therapies may contribute to effective therapies for IPF. Further studies with more detailed work are necessary to completely expose the molecular mechanisms behind the protective effect of naringin against bleomycin-induced pulmonary injury.

## Figures and Tables

**Table 1:** Effects of naringin on malondialdehyde (MDA), superoxide dismutase (SOD), and glutathione peroxidase (GPx) levels in lung tissues of bleomycin, naringin, and control groups.

Groups	MDA (nmol/g protein)	SOD (U/mg protein)	GPx (nmol/min/mg protein)
Control	20.83 ± 10.20	131.50 ± 5.61	10.33 ± 1.63
Bleomycin	65.00 ± 9.57^##^	91.83 ± 4.62^##^	18.66 ± 4.54^##^
Bleomycin + naringin 40	59.30 ± 9.64	99.33 ± 5.50	17.34 ± 2.42
Bleomycin + naringin 80	28.34 ± 7.94^*∗∗*^	113.83 ± 9.47^*∗*^	12.32 ± 2.65^*∗*^

^**##**^ *p* < 0.05, versus control group; ^*∗*^ *p* < 0.05, ^*∗∗*^ *p* < 0.01 versus bleomycin group. Data were expressed as “mean ± S.D.” for six rats in each group. Comparisons between the results for four groups were made by using one-way ANOVA followed by Tukey-Kramer multiple comparison tests.
